# A Comprehensive Overview of Vision Screening Programmes across 46 Countries

**DOI:** 10.22599/bioj.260

**Published:** 2022-06-10

**Authors:** Jill Carlton, Helen J. Griffiths, Paolo Mazzone, Anna M. Horwood, Frea Sloot, EUSCREEN Study Consortium

**Affiliations:** 1 School of Health and Related Research (ScHARR), University of Sheffield, UK; 2 Division of Ophthalmology & Orthoptics, Health Sciences School, University of Sheffield, UK; 3 University of Reading, UK; 4 Department of Ophthalmology, Erasmus Medical Centre, Rotterdam, NL

**Keywords:** vision screening, reduced vision, amblyopia, photoscreening, effectiveness

## Abstract

**Purpose:**

To describe and compare vision screening programmes and identify variance in number and type of tests used, timing of screening, personnel involved, monitoring and funding to be used as data for optimising, disinvesting or implementing future screening programmes.

**Methods:**

A questionnaire consisting of nine domains: demography & epidemiology, administration & general background, existing screening, coverage & attendance, tests, follow-up & diagnosis, treatment, cost & benefit and adverse effects was completed by Country Representatives (CRs) recruited from 47 countries.

**Results:**

The questionnaire was sufficiently completed for 46 Countries: 42 European countries, China, India, Malawi and Rwanda. Variation of provision was found in; age of screening (0–17 years), tests included (23), types of visual acuity (VA) test used (35 different optotypes), personnel (13), number of screens per child (median 5, range 1–32), and times VA tested (median 3, range 1–30). Infant screening is offered in all countries, whereas childhood vision screening is offered at least once in all countries, but not all regions of each country. All 46 countries provide vision screening between the ages of 3–7 years. Data on screening outcomes for quality assurance was not available from most countries; complete evaluation data was available in 2% of countries, partial data from 43%.

**Conclusion:**

Vision screening is highly variable. Some form of VA testing is being undertaken during childhood. Data collection and sharing should be improved to facilitate comparison and to be able to optimise vision screening programmes between regions and countries.

## Introduction

Screening is defined for the World Health Organisation (WHO) as ‘the presumptive identification of unrecognised disease or defect by the application of tests, examinations, or other procedures which can be applied rapidly. Screening tests sort out apparently well persons who probably have a disease from those who probably do not’ (Wilson & Junger, 1968; WHO, 2020). WHO criteria for an effective screening programme have been clearly outlined and supported for many decades (Wilson and Jungner, 1968). In a relatively recent review for the WHO, Andermann et al. (2008) found the Wilson and Jungner (1968) criteria to have stood the test of time, suggesting only minor modifications for emerging techniques and modern practices in medicine. The criteria are applied to include the entire process, including defining the test and referral criteria to identify the target condition, identifying the appropriate eligible population, ensuring access to effective treatment for individuals diagnosed with disease and monitoring the outcomes of the screening. Without this level of scrutiny, the most effective screening outcomes and use of limited resources cannot be achieved. Healthcare systems are facing increased demands (Burström, 2009; Hosseini, 2020), with increasing life expectancy and population growth contributing to stretched resources. It is therefore important to examine both existing and proposed interventions to ensure that they are not only clinically effective, but also cost-effective (Public Health England, 2015).

Childhood vision screening is a public health intervention that exists in many countries across the world, although the content of the vision screening programmes has been shown to vary tremendously both between and within countries. In a preliminary inventory of vision screening of 35 European countries, Sloot et al. (2015) found that programmes varied greatly in terms of the age at which screening is conducted (3–7 years), optotype charts used, referral criteria and professionals conducting vision screening. This provides great difficulty for policy makers to decide which vision screening programme to implement in countries where none exist or when reviewing current provision (Wang at al. 2019; Limburg et al. 1994). It may also indicate that effective and efficient screening systems are lacking in some countries. For this reason, the EUSCREEN study aimed to evaluate and compare the cost-effectiveness of screening programmes to enhance practice. Detailed (validated) data could be used as input for a microsimulation model that could be used by countries to optimise their screening programme (Verkleij et al, 2021). The purpose of this paper is to report the results from an international survey of childhood vision screening programmes, specifically evaluating existing approaches to vision screening to better understand the variability across programmes. This paper considers aspects of vision screening including number and type of tests used, timing of screening, professionals involved, monitoring and funding as outlined in WHO guidance, in relation to current European and wider international provision.

## Materials and Methods

The research followed the Tenets of the Declaration of Helsinki.

The Country-Committees Joint-Partnership of EUS€REEN Study Consortium was formed, an international collaboration of Country Representatives (CRs) with expertise and local knowledge in hearing, vision and general screening. This formed an international collaboration representing all countries associated with the Horizon 2020 programme (in 2014). Commencing January 2017, for each of these countries, CRs for vision screening were actively identified through screening organisations, publications on the subject in peer reviewed journals, national Ophthalmology and Orthoptic societies, existing professional contacts and through other CRs who had already registered. All CRs were contacted and recruited through e-mail, telephone calls and in-person during conferences.

### Development of the questionnaire

A questionnaire was developed to gather detailed information on general paediatric, vision and hearing screening programmes. The authors, with input from vision and screening experts currently practicing across several European countries, formulated, revised and agreed on 126 questions on vision screening. An additional 191 questions were formulated on hearing screening and 82 questions on general screening. The questionnaire consisted of nine domains containing information relating to: demography and epidemiology, administration and general background of the screening programme(s), existing screening systems, coverage and attendance, screening tests, follow-up and diagnostics, treatment availability, outcomes of screening and costs.

Three types of questions were used: open-ended, multiple-choice and yes-no questions. Most of the questions were followed by a sub-question about the source of the information provided. A respondent could choose between (a) Data unavailable, (b) I don’t know, (c) Rough estimate, (d) Real estimate from calculation, or (e) Actual data. The questionnaire then asked for the name and date of the data source if indicated.

### Data collection and validation

The questionnaire was distributed on a web-based platform, accessible through the EUSCREEN website – www.euscreen.org. (A copy of the questionnaire can be found here: https://www.euscreen.org/questionnaire/). CRs registered online and progressed through a tender procedure, in which their role in the local screening programme and access to data were assessed. Once accepted, the CR could log in using a unique username and password to complete the questionnaire in as many sessions as required before final submission. Completion and final submission of questionnaires was encouraged using weekly reminders. When CRs were unable to access the requested data, or lacked the time to fill out the questionnaire, measures were taken to recruit replacement or additional CRs. CRs were encouraged to complete the questionnaires to represent the entire country. Where regional differences occurred and information was unknown from other regions within a country, details of the CR’s known region was collected but noted as lacking representation of the entire country.

**Figure 1:**
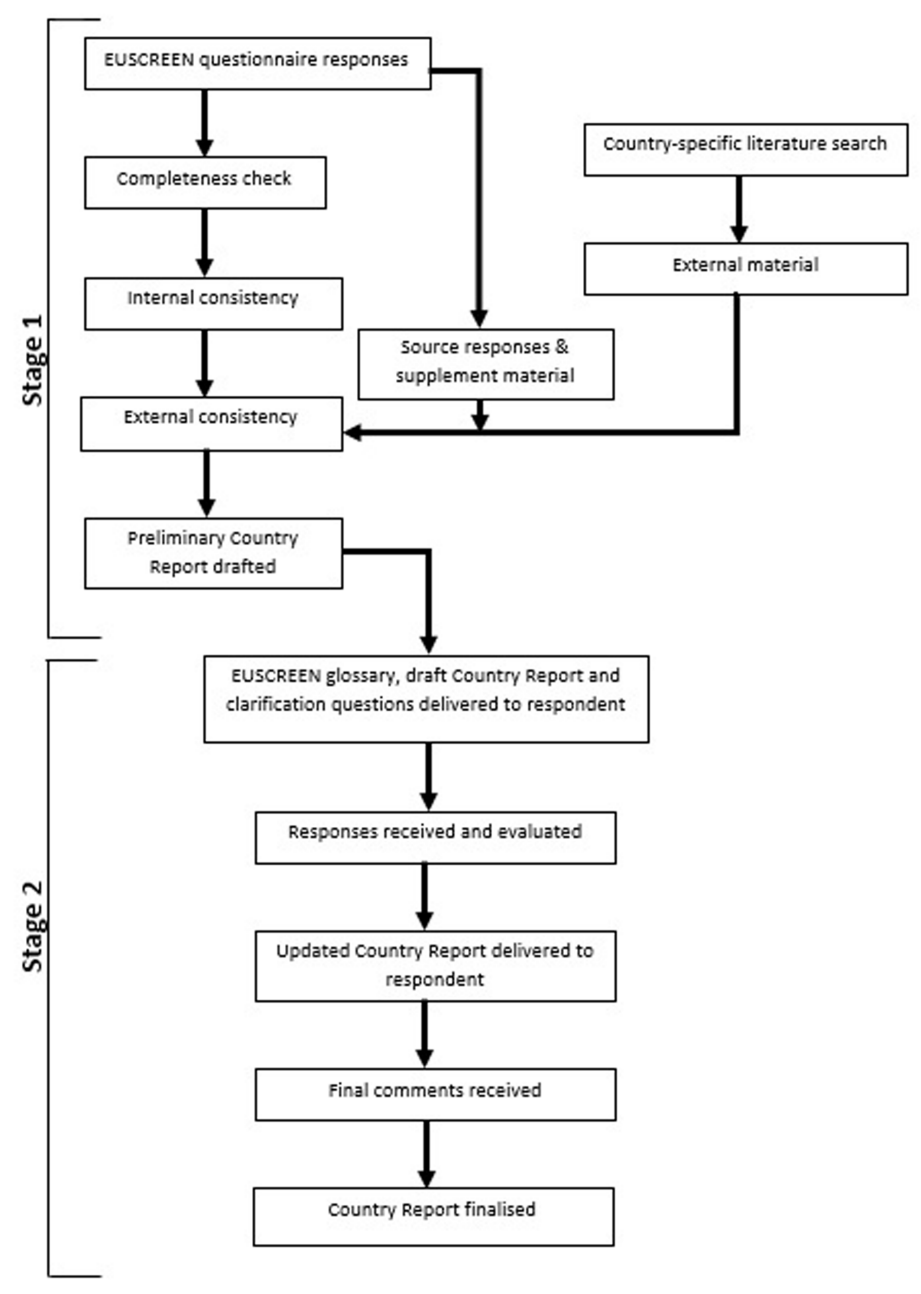
Flowchart of the verification process.

Existing vision screening provision across all participating countries was then documented. The process of documenting existing vision screening provisions across these countries involved several stages of validation to complete individual Country Reports. The verification process consisted of two stages as shown in Figure 1 and included the CRs attributing a numerical value to data reliability for each question (i.e., 0: Missing data to 5: Actual data with source and year). This was used to identify which country responses contained evidenced data and were most complete. These Country Reports could therefore be completed first to provide full version templates on which to base all other reports. Where conflicting information was provided from joint or collaborating CRs from the same country, clarification was sought via further questions incorporated into a draft report sent to the CRs. Further verification was completed by checking that the evidence submitted by CRs matched the interpretation given in the questionnaire. Additionally, country specific literature searches were completed to ensure matched responses and make any additions where information was missing.

Information was included in the reports regarding the geographic, demographic, economic and health situations in each country, as such information may inform choices made for implementation of screening programmes. The data was obtained from published, publicly available information (World Bank, 2019; World Health Organisation, 2016). Each draft Country Report followed the same template and contained sections relating to: the population and healthcare system; vision screening commissioning and guidance; vision screening procedure for each age group (premature babies to age 7 years); automated vision screening; provision for visually impaired; and knowledge of existing screening programmes (coverage; referrals; screening evaluation of true/false positives, sensitivity and specificity; and cost of vision screening).

The draft reports were returned to the relevant CRs between January and December 2018 for content validation checks. This step ensured that interpretation of the initial submitted responses to the questionnaire, supplementary material and external literature was verified. Further questions for clarification purposes were included within the draft report for the CR to answer. Additional questions were also added at this stage, relating to automated screening using automated photo-screening devices for detection of amblyopic risk factors, for countries where this was carried out. This relates to the practice of screening, typically earlier in childhood, for amblyogenic risk factors (for example, hypermetropia, astigmatism, anisometropia) rather than screening for the presence of reduced vision occurring when amblyopia has developed. The reason for additional questions relating to screening for risk factors was due to unforeseen details that were needed to populate and calibrate the model. Details of this additional information are reported elsewhere (Horwood et al. 2020). Any additional information provided by the CR was used to update and create a final report. The final reports were completed and sent to the CRs by 31^st^ December 2018 and any additional suggestions or changes implemented in the following three-month period. This allowed for a final stage of verification and content validation. Data from all countries was then compiled and compared for this report, CRs were asked to check all combined data tables to confirm correct interpretation in April 2021.

## Results

### General Country information

Completed vision screening questionnaires were submitted for 43 European countries and four other countries (China, India, Malawi and Rwanda) by November 2018. The EUSCREEN project was somewhat evolving in nature. Whilst the original application aimed to include EU countries, interest in the project grew more globally. To that end, four countries outside of Europe submitted data which is presented. One European country was omitted from the analysis due to insufficient data provided, leaving 46 countries included in the summary analysis. These countries were classified using the World Bank (2019) definitions as low income countries (LICs)—Malawi and Rwanda; lower-middle income countries (LMICs)—India, Kosovo and Moldova; upper-middle income countries (UMICs)—Albania, Bosnia and Herzegovina, Bulgaria, China, Macedonia, Montenegro, Romania, Serbia, and Turkey; and finally, high-income countries (HICs)—Austria, Belgium, Croatia, Cyprus, Czech Republic, Denmark, Estonia, England and Wales, Faroe Islands, Finland, France, Germany, Greece, Hungary, Iceland, Israel, Italy, Latvia, Lithuania, Luxembourg, Malta, Netherlands, Northern Ireland, Norway, Poland, Republic of Ireland, Scotland, Slovakia, Slovenia, Spain, Sweden and Switzerland. Final individual Country Reports were completed and published on the EUSCREEN website for 46 countries in March 2019 (Mazzone et al. 2019). The data was then combined to provide a summary of vision screening provision across the 46 countries or regions within countries.

### Organisation and funding

The organisation and funding of vision screening varies between and within countries. Funding of services ranges from national central government funding arrangements for universal screening programmes, to local government funding of regional service. These were reported in some countries or regions to be supplemented by additional screening funded through private health insurance or parental private payment.

Most countries (91%) reported provision of nationally organised and funded screening at birth for retinopathy of prematurity (ROP) when indicated and/or congenital ocular defects. With this national funding and organisation of infant vision screening a consistent approach was adopted across the country and all children were offered screening. In comparison, childhood screening for reduced vision or amblyopia was funded and organised nationally in 30 of the 46 (65%) countries, funded nationally but organised regionally in 2 (4%) countries, funded and organised regionally in 11 countries (24%), and through sporadic regional, charitable or research funding in 3 countries (7%). Nationally funded and organised childhood vision screening was delivered in none of the 2 LICs, 2 of the 3 LMICs, 5 of the 9 UMICs and 23 of the 32 (72%) HICs. Countries where regional organisation was in place (28%) had varying protocols in terms of eligible populations, screening professionals, tests, age groups screened, number of screens per child and referral criteria. Additionally, these countries had regions where no childhood screening for reduced vision or amblyopia is offered. Whilst all countries reported provision of screening for reduced vision in a least one region, in two (4%) countries (both UMICs) these were only temporary programmes offered with limited charitable funding in specific cities or villages (Romania and Bulgaria). In the 30 countries that had nationally funded and organised vision screening for reduced vision or amblyopia, 15 had a national protocol for testing, whereas the other 15 had varied testing protocols usually based on clinician’s choice.

### Screening professions

A total of 13 different screening professionals were reported to deliver vision screening across the 46 countries over all age groups. Table 1 provides details of the screening professionals delivering screening for each age group in each country. In some countries different screening professions provide screening between and within regions. Well babies born at full term are screened in 44 of the 46 countries for congenital ocular defects at birth, the exceptions being Rwanda, where no infant screening takes place, and Malawi, where screening is only offered in the southern region. Delivery of screening at this age was by 8 different professions, which varied both between and within countries as shown in Table 1. In the majority of countries, vision screening before 3 months of age is delivered by paediatricians (70%) or general practitioners (GPs) (46%). Alternative professionals conducting the screening ranged from nurses (30%), ophthalmologists (33%), neonatologists (13%) orthoptists (9%), health visitors (7%) and midwifes (2%).

**Table 1:** Professionals delivering vision screening.


	WELL, HEALTHY BABIES – BIRTH TO 3 MONTHS									BETWEEN 3 TO 36 MONTHS										BETWEEN 3 YEARS AND 7 YEARS									
		
	O	P	GP	N	OR	NN	HV	M		O	P	GP	N	OR	OPT	HV	MO	HCA		O	P	GP	N	OR	HV	OPT	HCA	CHD	T

Albania	✓	✓		✓						✓										✓	✓								

Austria		✓	✓							✓	✓	✓								✓	✓	✓		✓		✓			

Belgium		✓	✓							✓	✓	✓	✓	✓				✓			✓	✓	✓						

B&H	✓									✓										✓				✓					

Bulgaria						✓																	✓						

China	✓	✓								✓	✓									✓	✓								

Croatia		✓	✓								✓									✓							✓		

Cyprus	✓	✓								✓			✓	✓						✓				✓	✓	✓			

CR		✓		✓							✓		✓								✓		✓						

Denmark			✓	✓								✓	✓									✓	✓						

E&W		✓		✓																			✓	✓			✓		

Estonia			✓			✓						✓											✓						

FI			✓	✓								✓	✓									✓	✓						

Finland			✓	✓								✓	✓									✓	✓						

France	✓	✓	✓		✓					✓	✓	✓	✓	✓						✓	✓	✓	✓						

Germany		✓									✓										✓		✓				✓		

Greece	✓	✓								✓	✓									✓									

Hungary						✓	✓			✓						✓				✓					✓				

Iceland		✓	✓	✓							✓	✓	✓										✓						

India	✓	✓			✓	✓				✓				✓	✓					✓				✓		✓			✓

Israel		✓		✓										✓	✓								✓			✓			

Italy	✓	✓			✓						✓			✓						✓	✓			✓					

Kosovo	✓									✓										✓	✓	✓							

Latvia	✓	✓	✓			✓				✓	✓	✓								✓	✓	✓							

Lithuania	✓	✓		✓		✓				✓	✓	✓	✓							✓	✓	✓	✓						

Luxembourg		✓	✓											✓										✓					

Rep. Macedonia	✓									✓										✓									

Malta			✓	✓								✓	✓									✓	✓						

Malawi	✓	✓								✓										✓						✓			

Rep. Moldova	✓									✓	✓	✓								✓	✓	✓							

Montenegro		✓									✓										✓								

Netherlands			✓										✓										✓					✓	

NI		✓	✓				✓									✓							✓						

Norway		✓	✓	✓								✓	✓										✓						

Poland		✓	✓								✓	✓									✓	✓	✓						

ROI		✓	✓	✓									✓				✓						✓						

Romania		✓	✓																	✓									

Rwanda																							✓				✓		

Scotland			✓				✓																	✓					

Serbia		✓									✓									✓			✓	✓					

Slovakia		✓									✓										✓								

Slovenia		✓											✓				✓						✓				✓		

Spain		✓									✓										✓		✓			✓			

Sweden		✓	✓									✓	✓										✓						

Switzerland	✓	✓		✓	✓			✓		✓	✓	✓	✓	✓	✓					✓	✓	✓	✓	✓		✓			

Turkey		✓	✓							✓	✓	✓								✓	✓	✓							

**Total no of countries**	**15**	**32**	**21**	**14**	**4**	**6**	**3**	**1**		**18**	**20**	**17**	**16**	**8**	**3**	**2**	**2**	**1**		**21**	**18**	**15**	**24**	**10**	**2**	**7**	**5**	**1**	**1**


CHD = Child healthcare doctor GP = General Practitioner (family doctor) HCA = health care assistant (this includes school medical specialist, assistant nurse, orthoptic assistant) HV = health visitor MO = medical officer M = midwife N = nurse NN = neonatologist O = ophthalmologist Opt = optometrist or optician Or = orthoptist P = paediatrician T = teacher B&H = Bosnia & Herzegovina, CR = Czech Republic, E&W = England and Wales, FI = Faroe Islands, NI = Northern Ireland, ROI = Republic of Ireland

Five countries (11%) do not perform any vision screening of children aged 3 to 36 months (Bulgaria, England and Wales, Romania, Rwanda and Scotland). The other 41 countries offer screening within this age range within at least one region and one occasion, with 9 different health professionals delivering the screening as shown in Table 1. Paediatricians (43%), Ophthalmologists (39%), GPs (37%) and nurses (38%) were the most frequently used professions to deliver screening at this age. However, the level of eye or medical specific knowledge and expertise ranged from employment of highly trained experts, (i.e., ophthalmologists) as screeners (39%) to healthcare assistants (2%), who have no formal medical training.

All 46 countries provide vision screening between the age of 3 and 7 years in at least one region (although two of these countries services are provided by charities or research projects on a temporary basis) with a range of eleven different professionals involved in delivery as shown for each country in Table 1. The most frequently used profession is nurses (52%) but professions with more medical or eye-specific training were also employed for delivery of this task; Ophthalmologists (46%), GPs (33%), Paediatricians (39%), Orthoptists (22%). Vision screeners specifically trained for the task from general healthcare assistant (11% of countries) professionals were more frequently used in this older age group. India has employed the use of teachers for the role in the 3–7-year-old age group.

### Tests used, age and frequency of screening

Table 2 shows the number of eye screening interventions offered to each child in each country (or within regions of a country) in each age category (3 to 36 months, 3 to 7 years. Supplementary Table 1 also shows 7 to 17 years). Of these screening episodes the number which include a VA test is also shown for each country and age group. The final two columns of Table 2 show the total number of vision screening episodes offered across all age groups and the number of times VA specifically is tested in each child. The number of vision screening interventions ranges from 1 to 32 (median 5) with VA being measured between 1 and 30 times (median 3) in each child depending on the country or region(s) within countries.

**Table 2:** Frequency of Vision screening and Visual Acuity measurement delivered and optotype used for each age group.


AGE GROUP	BETWEEN 3 TO 36 MONTHS					BETWEEN 3 TO 7 YEARS					TOTAL NO OF VA SCREENS	TOTAL NO OF EYE SCREENINGS
		
FREQUENCY OF SCREENING AND VA TEST TYPE	NUMBER OF EYE SCREENINGS	NO OF VA SCREENS	PICTURE	NUMBER/SYMBOL	LETTER	NUMBER OF EYE SCREENINGS	NO OF VA SCREENS	PICTURE	NUMBER/SYMBOL	LETTER		

Albania *	1	1	•	•	•	2	2	•	•	•	3	3

Austria*	4	0		α		3	3		α		3	7

Belgium (Fl)*	2	0				3	3	•	•	•	3	5

Belgium (Fr)*	1	0				3	3	•	•	•	3	4

Belgium (G)*	1	0				3	3	•	•	•	3	4

Bosnia *	1	0				2	2	β	•		2	3

Bulgaria *	0	0				1	1		•		1	1

China	2	0				9	9	•	•	•	9	11

Croatia *~	3	0				2	2		•		2	5

Cyprus *	1	1	•			3	3	•		αβ	4	4

Czech Republic *	6	0				3	3	•	•	•	3	9

Denmark *	3	0				5	5	β		β	5	8

E&W *~	0	0				1	1			α	1	1

Estonia ~	2	0				2	2		•	•	2	4

Faroe Islands *~	3	0				5	5		β	β	5	8

Finland *~	3	0				5	4		β	β	4	8

France *~	3	1	•			2	2	•	•	•	3	5

Germany *~	4	0				3	3	ββ			3	7

Greece *~	1	0				2	2		•	•	2	3

Hungary *	2	1		αα		4	4		αα		5	6

Iceland*~	8	0				2	2		α	α	2	10

India	3	3	β	β	β	3	3		β	β	6	6

Israel*~	1	0				4	4		β		4	5

Italy*~	1	0				2	2	••	••		2	3

Kosovo	1	0				2	2			β	2	3

Latvia*	1	1	•			2	2		••		3	3

Lithuania*	1	1	••	••	••	1	1	•	•	•	2	2

Luxembourg*	1	1	•			3	3	•	•	•	4	4

Rep. Macedonia*	1	0				5	5	•	•	•	5	6

Malta*	2	0				3	3			ββ	3	5

Malawi	1	0				1	1			β	1	2

Rep. Moldova*	2	2	••	••	••	2	2	••	••	••	4	4

Montenegro*	1	0				1	1	β			1	2

Netherlands*	3	0				3	3	β	β		3	6

NI~	1	0				1	1			α	1	2

Norway*~	4	0				1	1		α		1	5

Poland*	1	0				5	5	••	••	••	5	6

ROI*~	2	0				2	1			α	1	4

Romania*	0	0				1	1		αα		1	1

Rwanda	0	0				1	1		α		1	1

Scotland~	0	0				1	1	α		α	1	1

Serbia	0	0				2	2		β		2	2

Slovakia*	2	0	•			2	2	•	•	•	2	4

Slovenia*~	3	0				5	5		••	••	5	8

Spain*~	1	0				2	2	β	••	••	2	3

Sweden*	4	0				3	3		β	β	3	7

Switzerland*	2	1	••	••	••	1	1	••	••	••	2	3

Turkey~	1	0				1	1		αα		1	2


* VA test choice varied by clinician, ~ National guidelines which include stated VA test(s) to be used, α = logMAR, β = Sn, • = undefined Sn/logMAR, underlined text = crowded, no underline = uncrowded. Belgium (Fl) = Flemish community, Belgium (Fr) = French community, Belgium (G) = German community, B&H = Bosnia & Herzegovina, CR = Czech Republic, E&W = England and Wales, FI = Faroe Islands, NI = Northern Ireland, ROI = Republic of Ireland.

All participating countries or regions within countries provide at least one measurement of VA within their screening programmes at some stage, but this was not universally offered to all children within each country. Many countries do not offer entire population vision screening and in some, it is sporadic in nature depending on availability of charitable funding.

The tests performed in each country vary greatly within each age group in terms of optotypes used for measurement of VA, the number of other diagnostic tests employed and the referral criteria. In some countries the VA test(s) used were identified in clear national guidance, indicated by the dash symbol (~) in column 1 of Table 2. National guidance was not consistent between countries. Alternatively, the choice of vision test was determined by clinician’s preference and test availability, indicated by the star symbol (*) in column 1 of Table 2. Although 19 of the 46 countries have national guidance to standardise the test optotype used, some of these screening programmes still had variation due to the allowance for clinicians to maintain their judgement to decide on the test choice. Table 2 also shows the type of optotypes used in each country as shown by the ticked cells indicating whether picture, number, symbol or letter tests are used. Also shown are whether these are crowded/linear tests or uncrowded/single optotypes, logMAR based tests, Snellen based tests or a mixture of logMAR and Snellen. The majority (83%) of programmes used crowded or linear optotypes, but use of standardised progression logMAR charts was less prevalent (26%). Despite widespread availability of ‘gold standard’ VA tests using crowded and logMAR progression tests, they are often not used as part of vision screening programmes. Use of only Snellen tests occurred in 40% of countries, with 30% of countries reporting variability in use of logMAR and Snellen based on factors such as the region, availability of tests and clinician’s preference.

The questionnaire asked CRs to list the tests used for assessment of VA. Thirty-five different tests were used across the 46 countries. Table 3 (and Supplementary Table 2) provides more detailed information on the specific ages that each type of VA screening intervention is offered to each child within a country or region(s) of that country.

**Table 3:** Age, frequency and test type used for visual acuity screening.


	AGE IN YEARS																TOTAL N OF VA SCREENS

	0		1		2		3		4		5		6		7		

Albania						•		•						•			3

Austria								α		α		α					3

Belgium (Fl)			•		•		•		•				•				5

Belgium (Fr)				•			•				•				•		4

Belgium (G)				•			•		•				•				4

Bosnia								•		•							2

Bulgaria								•									1

China							α	α	α	α	α	α	α	α	α	α	10

Croatia										•						•	2

Cyprus		•						•				α				β	4

Czech								•				•				•	3

Denmark								β		β		β		β		β	5

E&W										α							1

Estonia								•						•			2

Faroes								β		β		β		β		β	5

Finland										β		β		β		β	4

France						•		•		•							3

Germany										β		β		β			3

Greece									α			α				α	3

Hungary							α		α		α		α		α		5

Iceland										α				α			2

India			•		•		•		β		β		β		β		7

Israel								β		β				β		β	4

Italy								•						•			2

Kosovo								β						β			2

Latvia				•				•						•			3

Lithuania				•				•									2

Luxembourg							•	•		•		•					4

Rep. Macedonia								•	•	•		•		•			5

Malta								β				β				β	3

Malawi														β			1

Rep. Moldova				•		•		•								•	4

Montenegro														β			1

Netherlands								β		β		β					3

NI										α							1

Norway										α							1

Poland								•		•		•		•		•	5

ROI										α							1

Romania								α									1

Rwanda									α								1

Scotland									α								1

Serbia								β						β			2

Slovakia								•				•		•			3

Slovenia								•	•	•		•				•	5

Spain										•				•			2

Sweden										β	β			β			3

Switzerland							•	•									2

Turkey								α						α			2


Belgium (Fl) = Flemish community, Belgium (Fr) = French community, Belgium (G) = German community, B&H = Bosnia & Herzegovina, CR = Czech Republic, E&W = England and Wales, FI = Faroe Islands, NI = Northern Ireland, ROI = Republic of Ireland. *Key* – α = logMAR, β = Snellen, • = undefined Sn/logMAR, underlined text = crowded, no underline = uncrowded.

Whilst VA is used as the only childhood vision screening test in five countries (Albania, Bulgaria, England & Wales, Northern Ireland and Rwanda), further tests are included in screening programmes in the majority (89%) of countries or regions. These tests include limited further assessments, such as cover test and ocular movements, to fuller assessments of binocular vision, including stereotests. The tests used in each country to screen within each age group are shown in Tables 4A to 4C. From birth to 3 months (Table 4A), the number of tests employed varies between countries from 0 to 10. Between 3 and 36 months of age (Table 4B), the number of screening tests carried out varies from 0 to 15, with 5 countries offering no screening within this age group. At age 3 to 7 years (Table 4C), vision screening is completed in at least one region of each country using between 1 and 15 tests, with all countries completing a test of VA, as further detailed earlier in Tables 2 and 3.

**Table 4:** Tests used in vision screening interventions in each country.


	EI	RR	F	PR	PM	EM	H	RE	CT	ACT	VA	S	AU	AS	BIG	CV	OTH	TOTAL NO. OF TESTS

TABLE 4A:0–3 MONTHS																		

Albania	✓	✓																**2**

Austria	✓	✓																**2**

Belgium	✓	✓	✓	✓														**4**

Bosnia	✓	✓	✓	✓		✓	✓	✓										**7**

Bulgaria		✓																**1**

China								✓										**1**

Croatia	✓	✓	✓	✓	✓	✓	✓	✓										**8**

Cyprus	✓	✓	✓	✓														**4**

Czech	✓	✓	✓			✓												**4**

Denmark	✓		✓					✓	✓									**4**

E&W	✓	✓	✓	✓	✓	✓	✓		✓									**8**

Estonia	✓	✓																**2**

Faroes	✓	✓	✓		✓													**4**

Finland	✓	✓		✓	✓													**4**

France	✓	✓	✓	✓	✓	✓	✓											**7**

Germany	✓	✓	✓			✓												**4**

Greece	✓	✓	✓	✓	✓	✓	✓	✓										**8**

Hungary	✓	✓	✓	✓		✓	✓											**6**

Iceland	✓	✓	✓			✓			✓	✓								**6**

India	✓	✓	✓	✓	✓	✓	✓	✓	✓	✓								**10**

Israel	✓	✓	✓			✓												**4**

Italy	✓	✓				✓												**3**

Kosovo	✓	✓	✓	✓		✓												**5**

Latvia		✓				✓	✓		✓									**4**

Lithuania		✓																**1**

Luxembourg	✓	✓		✓			✓											**4**

Rep. Macedonia	✓	✓	✓	✓		✓		✓										**6**

Malta	✓	✓	✓			✓												**4**

Malawi	✓	✓																**2**

Rep. Moldova	✓	✓		✓														**3**

Montenegro	✓	✓	✓			✓												**4**

Netherlands	✓	✓	✓	✓		✓	✓	✓	✓									**8**

NI	✓	✓	✓															**3**

Norway	✓	✓	✓														✓*	**4**

Poland		✓	✓	✓														**3**

ROI	✓	✓	✓															**3**

Romania	✓	✓		✓	✓													**4**

Rwanda																		**0**

Scotland	✓	✓																**2**

Serbia	✓		✓	✓	✓	✓	✓											**6**

Slovakia	✓	✓	✓			✓												**4**

Slovenia	✓	✓	✓	✓	✓	✓												**6**

Spain	✓	✓		✓		✓												**4**

Sweden	✓	✓	✓	✓	✓	✓												**6**

Switzerland	✓		✓	✓				✓										**4**

Turkey	✓	✓		✓			✓											**4**

**No of countries**	**40**	**41**	**29**	**23**	**11**	**23**	**12**	**9**	**6**	**2**	**0**	**0**	**0**	**0**	**0**	**0**	**0**	

* General eye examination.																		

**TABLE 4B:3–36 MONTHS**																		

Albania											✓							**1**

Austria	✓	✓	✓	✓	✓	✓		✓	✓									**8**

Belgium	✓		✓	✓		✓	✓		✓		✓	✓	✓	✓	✓	✓		**12**

Bosnia	✓	✓	✓	✓		✓	✓	✓	✓	✓			✓					**10**

Bulgaria																		**0**

China	✓					✓	✓	✓	✓	✓				✓				**7**

Croatia	✓	✓	✓	✓	✓	✓	✓		✓	✓								**9**

Cyprus	✓	✓	✓	✓	✓	✓		✓	✓	✓	✓	✓	✓					**12**

Czech	✓		✓			✓								✓				**4**

Denmark	✓		✓					✓	✓									**4**

E&W																		**0**

Estonia	✓	✓	✓			✓	✓											**5**

Faroes	✓	✓	✓		✓		✓											**5**

Finland	✓	✓	✓		✓	✓	✓		✓									**7**

France	✓	✓	✓	✓	✓	✓	✓		✓	✓	✓	✓		✓		✓		**13**

Germany	✓	✓	✓	✓	✓	✓	✓							✓				**8**

Greece	✓	✓	✓	✓	✓	✓	✓	✓	✓	✓								**10**

Hungary	✓	✓	✓	✓		✓	✓		✓	✓	✓	✓	✓	✓	✓	✓		**14**

Iceland	✓	✓	✓			✓			✓	✓								**6**

India	✓	✓	✓	✓	✓	✓	✓	✓	✓	✓	✓	✓	✓			✓	✓**	**15**

Israel	✓		✓		✓		✓											**4**

Italy	✓	✓	✓			✓	✓	✓	✓									**7**

Kosovo	✓	✓	✓		✓	✓			✓	✓		✓	✓					**9**

Latvia	✓	✓				✓			✓		✓	✓					✓*	**8**

Lithuania	✓	✓	✓	✓	✓	✓	✓	✓	✓	✓	✓	✓	✓	✓		✓		**15**

Luxembourg	✓		✓	✓		✓	✓		✓	✓	✓	✓	✓	✓			✓***	**12**

Rep. Macedonia	✓	✓	✓	✓		✓		✓	✓									**7**

Malta	✓	✓	✓	✓	✓	✓	✓	✓	✓									**9**

Malawi	✓	✓	✓	✓		✓	✓	✓	✓	✓								**9**

Rep. Moldova	✓	✓	✓	✓	✓	✓	✓	✓	✓		✓	✓		✓		✓	✓**	**14**

Montenegro	✓	✓	✓			✓												**4**

Netherlands	✓	✓		✓		✓		✓	✓									**6**

NI	✓																	**1**

Norway	✓	✓	✓	✓			✓											**5**

Poland			✓				✓											**2**

ROI	✓		✓															**2**

Romania																		**0**

Rwanda																		**0**

Scotland																		**0**

Serbia	✓		✓	✓	✓	✓	✓		✓	✓		✓						**9**

Slovakia	✓		✓			✓												**3**

Slovenia	✓	✓	✓	✓	✓	✓	✓											**7**

Spain	✓	✓	✓	✓		✓	✓		✓									**7**

Sweden	✓	✓	✓	✓		✓	✓											**6**

Switzerland	✓	✓	✓		✓	✓	✓		✓	✓	✓	✓	✓	✓		✓		**13**

Turkey	✓	✓		✓		✓	✓											**5**

**No of countries**	**39**	**29**	**35**	**23**	**17**	**33**	**27**	**14**	**26**	**15**	**11**	**12**	**9**	**10**	**2**	**7**	**4**	

Underline denotes at private clinics only. * Visual fields and convergence; ** Retinoscopy; *** Convergence.																		

**TABLE 4C: 36 MONTHS TO 7 YEARS**																		

Albania											✓						✓*	**2**

Austria	✓						✓		✓		✓	✓	✓					**6**

Belgium	✓			✓		✓			✓		✓	✓	✓	✓	✓	✓		**10**

Bosnia	✓	✓	✓	✓	✓	✓	✓		✓	✓	✓	✓	✓			✓		**13**

Bulgaria											✓							**1**

China	✓			✓	✓	✓	✓	✓	✓	✓	✓	✓	✓	✓		✓		**13**

Croatia											✓							**1**

Cyprus	✓	✓	✓	✓	✓	✓	✓	✓	✓	✓	✓	✓	✓	✓		✓		**15**

Czech	✓					✓					✓			✓		✓		**5**

Denmark								✓			✓							**2**

E&W											✓							**1**

Estonia	✓	✓	✓			✓	✓				✓							**6**

Faroes							✓				✓	✓				✓		**4**

Finland	✓	✓	✓	✓	✓	✓	✓		✓	✓	✓							**10**

France	✓	✓	✓	✓	✓	✓	✓		✓	✓	✓	✓		✓		✓		**13**

Germany	✓			✓			✓				✓	✓		✓				**6**

Greece	✓	✓	✓	✓	✓	✓	✓	✓	✓	✓	✓					✓		**12**

Hungary	✓	✓	✓	✓		✓	✓		✓	✓	✓	✓	✓	✓		✓		**13**

Iceland	✓		✓			✓			✓	✓	✓	✓						**7**

India	✓	✓	✓	✓	✓	✓	✓	✓	✓	✓	✓	✓	✓			✓	✓**λ	**19**

Israel									✓		✓			✓				**3**

Italy	✓	✓				✓		✓	✓	✓	✓	✓						**8**

Kosovo	✓	✓	✓		✓	✓			✓	✓	✓	✓	✓					**10**

Latvia	✓	✓				✓	✓		✓		✓	✓					✓**	**9**

Lithuania	✓	✓	✓	✓	✓	✓	✓	✓	✓	✓	✓	✓	✓	✓		✓		**15**

Luxembourg	✓		✓	✓		✓	✓		✓	✓	✓	✓	✓	✓			✓****	**12**

Rep. Macedonia	✓	✓	✓	✓		✓		✓	✓		✓		✓			✓		**10**

Malta			✓		✓	✓			✓	✓	✓	✓				✓		**8**

Malawi	✓	✓	✓	✓		✓	✓		✓	✓	✓							**9**

Rep. Moldova	✓	✓	✓	✓	✓	✓	✓	✓	✓	✓	✓	✓		✓		✓	✓***	**18**

Montenegro	✓										✓							**2**

Netherlands	✓			✓		✓		✓	✓		✓							**6**

NI											✓							**1**

Norway	✓	✓									✓							**3**

Poland							✓				✓					✓		**3**

ROI											✓							**1**

Romania					✓	✓			✓	✓	✓			✓				**6**

Rwanda											✓							**1**

Scotland				✓		✓			✓		✓	✓						**5**

Serbia	✓	✓		✓	✓	✓	✓		✓	✓	✓	✓						**10**

Slovakia	✓	✓		✓		✓					✓							**5**

Slovenia	✓	✓	✓	✓	✓	✓	✓				✓							**8**

Spain	✓	✓	✓	✓	✓	✓			✓	✓	✓					✓		**10**

Sweden	✓						✓				✓							**3**

Switzerland	✓	✓	✓		✓	✓	✓		✓	✓	✓	✓	✓	✓		✓		**13**

Turkey	✓	✓	✓	✓			✓				✓							**6**

**No of countries**	**32**	**22**	**20**	**22**	**16**	**29**	**23**	**10**	**27**	**20**	**46**	**21**	**12**	**13**	**1**	**17**	**5**	


Underline denotes at private clinics only. * No specified; ** Visual fields and convergence; *** Retinoscopy, Bagolini Glasses, Worth Lights and Prism Cover Test (PCT); **** convergence; λ Retinoscopy, Bagolini Glasses, Worth Lights. B&H = Bosnia & Herzegovina, CR = Czech Republic, E&W = England and Wales, FI = Faroe Islands, NI = Northern Ireland, ROI = Republic of Ireland. ACT = alternating cover test AS = automated screening AR = autorefraction BiG = Biprism of Gracis CT = cover test CV = colour vision EI = eye inspection EM = eye motility F = fixation H = Hirschberg Oth = other PM = pursuit movements PR = pupillary reflexes RE = retinal examination RR = red reflex S = stereopsis VA = visual acuity

In at least one region, 17 countries (37%) screen for colour vision defects, all between ages 3 to 7 years and seven of these countries also screen colour vision at age 3–36 months. Use of autorefractors or photoscreening devices to screen for amblyogenic risk factors in the 3 to 36-month age group occurs in 14 (30%) countries and between 3 and 7 years in 18 (39%) countries, although some of these regions or countries require private funding to receive this screening test intervention (see Table 4). Many CRs reported that specific protocols were not in place for these additional or alternative tests and the choice of tests is decided through clinical judgement, professional opinion and/or clinician preferences.

### Outcome data

Audit data was rarely available to the CRs, as it was rarely collected or was audited centrally and not accessible. There is limited evidence relating to the outcomes of the vision screening carried out in the 46 countries for which questionnaires were analysed. This was despite the best efforts of CRs who were well placed to be able to access available data. Only one country submitted complete prevalence data for the four categories of amblyopia (treated or untreated amblyopia, persistent amblyopia, strabismus and cataract) with 20 countries providing no data for any of the categories. Just 17 of the 46 (37%) countries provided information on coverage, for either national or regional programmes. Only one (2%) country submitted complete screening evaluation data (i.e., coverage, number of false positives, false negatives, sensitivity, specificity and positive predictive values), with 20 (43%) providing partial data and 25 (54%) providing no data for any of these categories.

Only two countries have complete data on the percentage of children treated for amblyopia, strabismus and cataract, 4 countries provided partial data and 39 countries (85%) were unable to provide any data in relation to numbers receiving treatment.

## Discussion

This survey confirmed the (major) variation found in the preliminary study by Sloot et al. (2015). In the current study the questionnaire was expanded, more countries were included, data were validated and checked with available literature, all of which make our conclusions more robust. Vision screening varies within and between countries in respect to funding, organisation, screening professionals, tests, frequency and ages screened. A total of 13 different professions were reported to deliver vision screening across the 46 countries over all age groups. Most countries had different professions delivering the screening at the same age across or within regions. For well babies born at full term, eight different medical professionals, most employed medically trained doctors (paediatricians, GPs, ophthalmologists or neonatologists) for this role, whilst in some programmes, personnel with nursing backgrounds or orthoptists delivered the infant screening. At 3 to 36 months, 9 different health professionals delivered screening, the majority still being highly medically trained (paediatricians, GPs and ophthalmologists) and hence more expensive. Screening professionals with nursing backgrounds were increasingly used to deliver screening at 3–36 months (38%), and in one country, the use of less qualified, trained screeners was employed (healthcare assistants). In the 3 to 7-year age group, the most frequently used screening professional were qualified nurses (52%), but professions with more medical or eye specific training were also employed for this task (ophthalmologists 40%, GPs 38%, paediatricians 36%). At this more co-operative age (i.e., 3–7 years) there were still only a small number of countries that employed trained vision screeners from general healthcare assistant staff for this task (7%). This wide range of personnel may reflect availability of staff across countries. Screening evaluation data and analysis with our cost-effectiveness model (miscan.euscreen.org) is required to determine if the reduced costs, where less expert personnel are used, can be successfully applied without reduction in effectiveness and outcomes.

The number of eye screening interventions offered to each child across countries varied, with measurement of VA offered between 1 and 30 times. There are regions within countries that only offer vision screening of infants and not of older children; therefore, VA may never be measured in some children. The tests performed vary greatly in terms of optotypes used for measurement of VA, the number of other diagnostic tests employed and the referral criteria. Whilst some countries or regions offered VA testing only, others included one or several additional tests such as cover test, ocular movements or assessment of corneal reflections, binocular vision tests including stereotests, colour vision tests and automated refraction. Thirty-five different VA tests were used across the 46 countries, the majority (83%) crowded or linear optotypes but more discriminative standardised progression logMAR charts (Bailey & Lovie, 1976; Hussain et al., 2006) were used in only 26%. Some of the VA tests reported do not have age-specific normative data sets making referral criteria in a screening scenario difficult to set.

Effectiveness of vision screening programmes and evaluation of the most effective protocols was not possible as only 17 countries were able to provide information on coverage and only one country was able to provide data on coverage, number of false positives, false negatives, sensitivity, specificity and positive predictive values, and 54% provided no data for any of these categories.

Our study was limited by the difficulty in obtaining data from respondents that represented the entire country or being sure of how many regions within a country it might relate to. We attempted to address regional differences and to validate and cross-check data, but CRs did not have access to country-wide information. Obtaining accurate information on funding and coverage was difficult and not possible in the majority of countries. Many of the vision screening programmes evolved without robust evidence on cost-effectiveness being available and without consideration of WHO criteria to design programmes with this in mind. The lack of regionally and nationally audited programmes, and hence absence of data, prevents further analysis of individual programme effectiveness and cost-effectiveness to determine a template for decision-makers and health planners. The principle that screening programmes should respond to a recognised need and be designed for a specific target condition(s) necessitates knowledge of the local prevalence of conditions and that the relative harm of conditions is considered. This ensures that screening target conditions are just those that are prevalent, cause a significant deficit and if left undiagnosed cannot be treated as successfully as they can following early identification. For example, reduced vision in childhood caused by amblyopia, has a prevalence in industrialised nations of 2–5% (Solebo et al. 2015), will most likely go undetected (as usually in just one eye) and results in irreversibly poor vision and an avoidable risk for subsequent visual impairment resulting from loss of vision in the non-amblyopic eye. It can be treated effectively, but the window for successful treatment is limited by the critical period. These characteristics identify amblyopia as a valid target condition for timely childhood screening. Other ocular conditions in childhood, such as strabismus, reduced convergence, ocular motility disorders, refractive error, reduced/absent stereopsis, or colour vision defects may not be considered to have these crucial factors needed to warrant costly (both financially and in parental time and anxiety) screening interventions (Sloot et al. 2021). Refractive error, which does not reduce VA sufficiently for a child to fail a VA screen, is a topic of current debate, as it fulfils fewer of the WHO criteria (i.e., not preventable, early treatment still of equivocal societal benefit).

The data collected from the EUSCREEN questionnaire revealed widespread lack of consideration of prevalence and the nature of specific target condition(s). For example, the age at which screening is offered (and often repeated throughout childhood) lacks specific effective condition-based targeting of resources. Tests employed often consist of a complete assessment of motility and binocular vision without evidence of detailed consideration of why adding a test to the ‘screening’ protocol for the detection of that deficit is of benefit. The wide difference in expenditure per screen and per child relate to the wide variation in the number of tests employed at each screening intervention (e.g., 1 to 17 tests per screen at age 3–7 years) and the frequency of vision screening offered to each child (1 to 13 screening interventions).

A vital aspect, when considering implementation or review of screening, is availability of scientific evidence to confirm the effectiveness of the proposed or current screening programme. It was evident from submitted data that information relating to coverage, number referred, true/false positives, specificity and sensitivity was not available. It is possible that data is being collected, but lack of regional and national publication or accessibility of the data prevents benchmarking, learning and development of actions to implement the most effective screening programmes. Currently it is impossible to evaluate the effectiveness of most vision screening programmes conducted within Europe. Validation of personnel delivering screening, the training processes and any need for retraining cannot be determined in most programmes. This absence of available data provides a barrier to ensuring effective clinical practice, avoidance of risk and harm and ensuring effective resource allocation. There should be quality assurance, with programme evaluation planned from the outset to minimise potential risks of screening. Such procedures would be advised at local levels for each programme, but ideally operated at a regional or national level to enable bench-marking and improvement in service standards through this process and sharing experience, methods and outcomes.

It is of concern that regional variation exists within most countries. Any programme should promote equity and access to screening for the entire target population (EU Council 2011; Cloete et al. 2019). Poor national data availability meant that some CRs could only report for specific regions. These regional variations produce an issue concerning how representative the data is of each entire country. Lack of provision of vision screening in the form of a VA test for all children was evident in both the highest income and lower income countries, whilst some countries or regions within countries were delivering this on multiple occasions. This inequity in the use of resources is placing some children at risk of visual impairment. Parents or carers rarely suspect reduced vision and/or amblyopia, unless the child has an associated large strabismus (Polling et al. 2012; Sloot et al. 2021). This particularly applies to the most vulnerable children in societies, where parents may lack the education or resources to seek or pay for private assessment when vision screening is unavailable.

A limitation within the data set includes problems with data reliability, particularly evidenced where more than one CR submitted questionnaire responses. The answers were regularly in conflict with each other. Further questions for clarification purposes were sent to both CRs to validate and verify the answers provided. Whilst some countries submitted empirical evidence from published sources, the majority of the data provided were based on professional estimates. This has led to discrepancies. Audit data is likely to be available at a local or regional level, but CRs either did not have access to it or did not know about it. This highlights the lack of databases and central resources to collect and standardise this process. Data governance with public access is needed to scrutinise, evaluate and share best practice.

The variation between screening programmes, such as tests conducted and professionals conducting screening, provides great difficulty for policy makers to decide which vision screening programme to implement in countries or regions where none exist. It also means that, effective and efficient screening systems are lacking with potential wasted resources in some instances. Similar to the findings of Sloot et al. (2015), the limited quality assurance reporting processes and limited information provided by CRs make it difficult to determine which vision screening programmes align themselves with the proposed screening criteria by Andermann et al. (2008). It is clear that most vision screening programmes require more rigorous methods of documentation, data collection, evaluation and public reporting. This will help ensure that children are receiving the best care, that the programmes are both clinically and cost-effective, and that adequate training can be provided for future vision screening professionals to manage, update and promote equity of care across the target population.

### Recommendations for the future

The known variation between countries documented by Sloot et al. (2015) informed the need for the EUSCREEN study. The current, more detailed investigation including more countries within Europe five years later, reveals the widespread variance in tests used (12 VA tests in Sloot et al., 35 in current study) and number of screening interventions (1 to >3 in Sloot et al, 1–32 in current study), and highlights further the lack of reported outcomes, providing evidence for the urgent need to review cost-effectiveness. It is still not possible to identify the most clinically and cost-effective vision screening programme for countries in Europe. This is due to the low level of reporting, documenting and research concerning the vision screening programmes found in each country, evidenced by the paucity of reliable, validated data available to and provided by CRs. We would encourage careful consideration of two critical components related to WHO criteria. Firstly, a clearly defined target condition(s) for screening. This will help determine what tests and referral criteria are included within the screening programme. Tests should be relevant for the target condition with consideration of sensitivity and specificity of the test to the target condition. Amblyopia is commonly quoted as the target for screening because of the critical period, but often what vision screening detects is mainly refractive error. Refractive error in children can only be confirmed with a full cycloplegic refraction, so not screening. Vision screening using a VA or orthoptic tests, and even photoscreening, only look for markers for significant refractive error which still needs to be confirmed. A subsequent consideration is how much difference does the correction of refractive error in early childhood make to a child’s life if their VA is adequate (but perhaps not perfect) (Horwood et al., 2021). Secondly, referral criteria (i.e., pass/fail criteria) should be clearly defined for the target condition(s), with consideration of age-appropriate normative data. The data collected as part of this study has highlighted that some screening programs contain many tests, meaning that the screening episode is longer (c.f. a single test). Consideration of what additional benefit a battery of tests has should be given. Whilst it may increase the sensitivity and specificity of the screening program itself, are the gains of value given that the consequence of longer screening episodes will mean less children can be screened in one day?

It is encouraging that screening for congenital eye disorders occurs in most of the countries described. This has been shown to be very cost-effective (van den Akker-van Marle et al. 2015). Furthermore, some form of VA testing is being undertaken during childhood. There is little doubt that children with poor vision should be identified; and some form of screening is indicated, but this study highlights the lack of international consensus and very poor availability of data. This lack of data means that decisions cannot be based on evidence and sharing of best practice cannot occur.

## Additional Files

The additional files for this article can be found as follows:

10.22599/bioj.260.s1 Supplementary Table 1. Frequency of Vision screening and Visual Acuity measurement delivered and optotype used for each age group.

10.22599/bioj.260.s2 Supplementary Table 2. Age, frequency and test type used for visual acuity screening.
