# Supplementary material for: A Comprehensive Overview of Vision Screening Programmes across 46 Countries
Source: Br Ir Orthopt J. 2022 Jun 10;18(1):27–47. doi: 10.22599/bioj.260 (PMC9187246; doi:10.22599/bioj.260)
Supplement: Supplementary Table 1. — Frequency of Vision screening and Visual Acuity measurement delivered and optotype used for each age group. [file bioj-18-1-260-s1.pdf]

SMT Table 1: Frequency of Vision screening and Visual Acuity measurement delivered and optotype used for each age group.

* VA test choice varied by clinician, ~ National guidelines which include stated VA test(s) to be used, Red text = logMAR, Green text = Sn, Blue text = undefined Sn/logMAR, bold text = crowded, un-bold = uncrowded

**Age group**	**Between 3 to 36 months**					**Between 3 to 7 years**					**Between 8 to 17 years**						
Frequency of screening and VA test type	Number of eye screenings	No of VA screens	Picture	Number/Symbol	Letter	Number of eye screenings	No of VA screens	Picture	Number/Symbol	Letter	Number of eye screenings	No of VA screens	Picture	Number/Symbol	Letter	**Total no of VA screens**	**Total no of eye screenings**
Albania *	1	1	✓	✓	✓	2	2	✓	✓	✓	0	0				3	3
Austria*	4	0		✓		3	3		✓		0	0			✓	3	7
Belgium (Fl)*	2	0				3	3	✓	✓	✓	4	4		✓	✓	7	9
Belgium (Fr)*	1	0				3	3	✓	✓	✓	5	5		✓	✓	8	9
Belgium (G)*	1	0				3	3	✓	✓	✓	4	4		✓	✓	7	8
Bosnia *	1	0				2	2	✓	✓		0	0				2	3
Bulgaria *	0	0				1	1		✓		0	0				1	1
China	2	0	✓	✓	✓	9	9	✓	✓	✓	21	21	✓	✓	✓	30	32
Croatia *~	3	0				2	2		✓		0	0				2	5
Cyprus *	1	1	✓			3	3	✓		✓✓	0	0				4	4
Czech Republic *	6	0				3	3	✓	✓	✓	4	4	✓	✓	✓	7	13
Denmark *	3	0				5	5	✓		✓	0	0				5	8
E&W *~	0	0				1	1			✓	0	0				1	1
Estonia ~	2	0				2	2		✓	✓	0	0				2	4
Faroe Islands *~	3	0				5	5		✓	✓	1	1		✓	✓	6	9
Finland *~	3	0				5	4		✓	✓	2	2		✓		6	10
France *~	3	1	✓			2	2	✓	✓	✓	0	0				3	5
Germany *~	4	0				3	3	✓✓			0	0				3	7
Greece *~	1	0				2	2		✓	✓	5	5		✓✓	✓✓	7	8
Hungary *	2	1		✓✓		4	4		✓✓		4	4		✓✓		9	10
Iceland*~	8	0				2	2		✓	✓	3	3		✓	✓	5	13

Age group	Between 3 to 36 months					Between 3 to 7 years					Between 8 to 17 years					Total no of VA screens	Total no of eye screenings
Frequency of screening and VA test type	Number of eye screenings	No of VA screens	Picture	Number/Symbol	Letter	Number of eye screenings	No of VA screens	Picture	Number/Symbol	Letter	Number of eye screenings	No of VA screens	Picture	Number/Symbol	Letter		
India	3	3	✓	✓	✓	3	3		✓	✓	11	11		✓	✓	17	17
Israel*~	1	0				4	4		✓		0	0				4	5
Italy*~	1	0				2	2	✓✓	✓✓		0	0				2	3
Kosovo	1	0				2	2			✓	2	2			✓	4	5
Latvia*	1	1	✓			2	2		✓✓		0	0				3	3
Lithuania*	1	1	✓✓	✓✓	✓✓	1	1	✓	✓	✓	0	10			✓	12	2
Luxembourg*	1	1	✓			3	3	✓	✓	✓	6	6		✓		10	10
Rep. Macedonia*	1	0				5	5	✓	✓	✓	0	0				5	6
Malta*	2	0				3	3			✓✓	0	0				3	5
Malawi	1	0				1	1			✓	0	0				1	2
Rep. Moldova*	2	2	✓✓	✓✓	✓✓	2	2	✓✓	✓✓	✓✓	0	0				4	4
Montenegro*	1	0				1	1	✓			0	0				1	2
Netherlands*	3	0				3	3	✓	✓		0	0				3	6
NI~	1	0				1	1			✓	0	0				1	2
Norway*~	4	0				1	1		✓		0	0				1	5
Poland*	1	0				5	5	✓✓	✓✓	✓✓	0	0				5	6
ROI*~	2	0				2	1			✓	0	0				1	4
Romania*	0	0				1	1		✓✓		0	0				1	1
Rwanda	0	0				1	1		✓		0	0				1	1
Scotland~	0	0				1	1	✓		✓	0	0				1	1
Serbia	0	0				2	2		✓		1	1			✓	3	4
Slovakia*	2	0	✓			2	2	✓	✓	✓	0	0		✓	✓	2	4
Slovenia*~	3	0				5	5		✓✓	✓✓	0	0				5	6
Spain*~	1	0				2	2	✓	✓✓	✓✓	1	1		✓✓	✓✓	3	4
Sweden*	4	0				3	3		✓	✓	0	0				3	7

Age group	Between 3 to 36 months					Between 3 to 7 years					Between 8 to 17 years					Total no of VA screens	Total no of eye screenings
Frequency of screening and VA test type	Number of eye screenings	No of VA screens	Picture	Number/Symbol	Letter	Number of eye screenings	No of VA screens	Picture	Number/Symbol	Letter	Number of eye screenings	No of VA screens	Picture	Number/Symbol	Letter		
Switzerland*	2	1	✓✓	✓✓	✓✓	1	1	✓✓	✓✓	✓✓	0	0				2	3
Turkey~	1	0				1	1		✓✓		0	0				1	2

Belgium (Fl) = Flemish community, Belgium (Fr) = French community, Belgium (G) = German community, B&H = Bosnia & Herzegovina, CR = Czech Republic, E&W = England and Wales, FI = Faroe Islands, NI = Northern Ireland, ROI = Republic of Ireland
