# Supplementary material for: A Comprehensive Overview of Vision Screening Programmes across 46 Countries
Source: Br Ir Orthopt J. 2022 Jun 10;18(1):27–47. doi: 10.22599/bioj.260 (PMC9187246; doi:10.22599/bioj.260)
Supplement: Supplementary Table 2. — Age, frequency and test type used for visual acuity screening. [file bioj-18-1-260-s2.pdf]

SMT Table 2: Age, frequency and test type used for visual acuity screening

	Age in years																																					Total N of VA screens
	0	1		2		3		4		5		6		7		8		9		10		11		12		13		14		15		16		17		18		
Albania					1		1						1																									3
Austria							1		1		1																											3
Belgium (Fl)		1		1		1		1				1				1				1				1				1										9
Belgium (Fr)			1			1				1				1				1				1		1		1				1								9
Belgium (G)			1			1		1				1				1				1				1				1										8
Bosnia							1		1																													2
Bulgaria							1																															1
China						1	1	1	1	1	1	1	1	1	1	1	1	1	1	1	1	1	1	1	1	1	1	1	1	1	1	1	1	1	1	1	1	32
Croatia									1						1																							2
Cyprus	1						1				1				1																							4
Czech							1				1				1								1				1				1				1			7
Denmark							1		1		1		1		1																							5
E&W									1																													1
Estonia							1						1																									2
Faroes							1		1		1		1		1																							5
Finland									1		1		1		1								1						1									6
France					1		1		1																													3
Germany									1		1		1																									3
Greece								1			1				1				1				1						1						1			7
Hungary						1		1		1		1		1		1				1				1				1										9
Iceland									1				1						1						1				1									5
India		1		1		1		1		1		1		1		1		1		1		1		1		1		1		1		1		1		1		18
Israel							1		1				1		1																							4
Italy							1						1																									2
Kosovo							1						1								1								1									4
Latvia			1				1						1																									3
Lithuania			1				1									1		1		1		1		1		1		1		1		1		1				12
Luxembourg						1	1		1		1						1				1				1				1				1				1	10
Rep. Macedonia							1	1	1		1		1																									5
Malta							1				1				1																							3
Malawi													1																									1
Rep.			1		1		1								1																							4

	Age in years																																					Total N of VA screens
	0	1		2		3		4		5		6		7		8		9		10		11		12		13		14		15		16		17		18		
Moldova																																						
Montenegro													1																									1
Netherlands							1		1		1																											3
NI									1																													1
Norway									1																													1
Poland							1		1		1		1		1																							5
ROI									1																													1
Romania							1																															1
Rwanda										1																												1
Scotland									1																													1
Serbia							1						1														1											3
Slovakia							1				1		1																									3
Slovenia							1	1	1		1				1																							5
Spain									1				1												1													3
Sweden									1	1			1																									3
Switzerland						1				1																												2
Turkey							1						1																									2

Belgium (Fl) = Flemish community, Belgium (Fr) = French community, Belgium (G) = German community, B&H = Bosnia & Herzegovina, CR = Czech Republic, E&W = England and Wales, FI = Faroe Islands, NI = Northern Ireland, ROI = Republic of Ireland

Key - 1 denotes number of VA screening episodes within the highlighted time period

	logMAR, crowded
	logMAR, uncrowded
	Sn, crowded
	Sn, uncrowded
	Undefined Sn/logMAR, crowded
	Undefined Sn/logMAR, uncrowded
